# Long-Term Results of Segmentectomy vs. Lobectomy for c-Stage IA Lung Cancer: A Real-Life Study with a Propensity Score Analysis Based on a National Cohort

**DOI:** 10.3390/jcm14072267

**Published:** 2025-03-26

**Authors:** Iker Lopez, Borja Aguinagalde, Juan A. Ferrer-Bonsoms, Laura Sánchez, Fernando Ascanio, Julio Sesma, José Luis Recuero, Arantza Fernandez-Monge, Jon A. Lizarbe, Raul Embun

**Affiliations:** 1 Department of Thoracic Surgery, Donostia University Hospital, 20014 San Sebastián-Donostia, Spain; borja.aguinagaldevaliente@osakidetza.eus (B.A.); arantza.fernandez-mongeumaran@osakidetza.eus (A.F.-M.); jonander.lizarbebon@osakidetza.eus (J.A.L.); 2 Lung and Pleural Diseases Research Group, Biogipuzkoa Health Research Institute, 20014 San Sebastián-Donostia, Spain; 3 Department of Biomedical Engineering and Science, School of Engineering (TECNUN), University of Navarra, 20018 San Sebastián-Donostia, Spain; jafhernandez@unav.es; 4 Department of Thoracic Surgery, Marqués de Valdecilla University Hospital, 39008 Santander, Spain; lasanchez@humv.es; 5 Department of Thoracic Surgery, Vall d’Hebron University Hospital, 08035 Barcelona, Spain; fernando.ascanio@vallhebron.cat; 6 Department of Thoracic Surgery, General de Alicante University Hospital, 03550 Alicante, Spain; jsesmaromero@gmail.com; 7 Thoracic Surgery Department, Miguel Servet University Hospital, IIS Aragón, 50009 Zaragoza, Spain; jlrecuero@salud.aragon.es (J.L.R.); raulembun@gmail.com (R.E.)

**Keywords:** lung cancer, segmentectomy, lobectomy, sublobar resection, recurrence, survival

## Abstract

**Background/Objectives**: The objective was to compare the results of segmentectomy and lobectomy in the treatment of c-stage IA lung cancer in terms of tumor recurrence and 5-year survival. **Methods:** An observational study was performed using 3533 patients included in the registry of the Spanish VATS Group (GEVATS) of the Spanish Society of Thoracic Surgery (SECT) between 2016 and 2018. A total of 1004 lobectomies and 83 segmentectomies in c-stage IA were selected. Two comparable groups were selected through 2:1 propensity score matching with patient-, tumor- and surgery-related variables, leaving 166 lobectomies and 83 segmentectomies. Tumor recurrence was analyzed by Fisher’s test and overall, cancer-specific, recurrence-free and disease-free survival by Kaplan-Meier and Log-rank tests. **Results:** Overall recurrence was 23.7% in both groups, with a predominance of locoregional recurrence in segmentectomy (16.2% vs. 11.2%) and distant recurrence in lobectomy (12.5% vs. 7.5%). There was no difference between the two groups in any of the survival types. Overall survival at 5 years was 73.5% (95% CI: 65.5–82.4%) in the lobectomy group vs. 73.1% (95% CI: 60.1–88.9%) in the segmentectomy group. **Conclusions:** Anatomic segmentectomy may be a valid option in the treatment of c-stage IA lung cancer since the recurrence and long-term survival outcomes compared to lobectomy are equivalent.

## 1. Introduction

The minimal lung resection type considered optimal in the surgical treatment of lung cancer has been lobectomy since the clinical trial published in 1995 by Ginsberg, which showed a greater locoregional recurrence in sublobar resections [1]. In older, frail patients or those with impaired respiratory function tests, sublobar resection has been a valid option in early stages [2,3]. It has also been valid for tumors with a predominance of ground glass [4]. Following observational studies with similar results between segmentectomy and lobectomy [5,6,7], two randomized clinical trials have promoted segmentectomy for the treatment of solid tumors smaller than 2 cm [8,9]. However, doubts persist due to the observation of a greater locoregional recurrence in segmentectomy [8].

The aim of our study was to compare the results of segmentectomy and lobectomy in the treatment of c-stage IA lung cancer in terms of tumor recurrence and 5-year survival, matched by propensity score. Data from the prospective cohort of the Spanish Group of Video-Assisted Surgery (GEVATS) were used [10].

## 2. Materials and Methods

The recommendations of the STROBE (STrengthening the Reporting of OBservational studies in Epidemiology) statement have been followed for the presentation of the article (available at STROBE—Strengthening the reporting of observational studies in epidemiology: https://strobe-statement.org/).

### 2.1. Ethical Statement

The initial GEVATS study was approved by the Clinical Research Ethics Committees of all the participating centers, in accordance with Law 14/2007 on Biomedical Research, the ethical principles of the Declaration of Helsinki and other applicable ethical principles. Patients provided informed written consent for their inclusion in the study and publication of their study data.

### 2.2. Study Design

An observational study was performed using data from the prospective GEVATS registry. The GEVATS project of the Spanish Society of Thoracic Surgery (SECT) was created to study the implementation of a video-assisted surgical approach (VATS) in our country, as well as to compare short- and long-term results between VATS and open surgery. A prospective multicenter cohort study was designed to include all anatomic lung resections performed in 33 Spanish centers (20 December 2016–20 March 2018). A follow-up was completed after 5 years in 30 centers, ending July 2022 (mean follow-up: 51.4 months). All the details about the characteristics of the database, the audit procedures and the definition of the variables are explained in the general publication of the GEVATS study [10].

### 2.3. Patients

**Figure 1:**
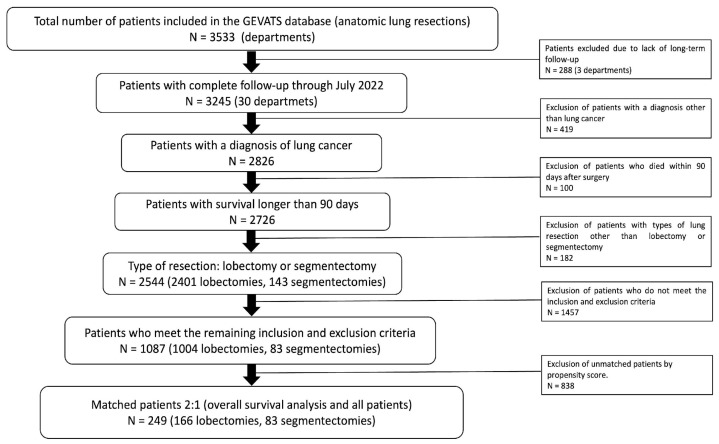
Flow chart of the selection of patients included in the study from the GEVATS database.

Patients who received a segmentectomy or lobectomy operation for non-small cell lung cancer, completed long-term follow-up and exceeded 90 days postoperatively were selected. The inclusion and exclusion criteria aimed to obtain a population similar to the previously mentioned clinical trials. Patients with stage cT1a-cN0, solid or mixed nodule in computed tomography (CT) and complete resection (R0) were included. Patients with pure ground glass tumors, neoadjuvant treatment, previous history of lung neoplasia, synchronous tumors or incomplete resection were excluded (Figure 1).

### 2.4. Variables and Outcomes

Patient-, tumor- and surgery-related variables were used in the descriptive analysis of the patients included in the study, and for propensity score matching.

Outcome variables were used to study the primary endpoints, which were tumor recurrence and survival. The frequency of recurrence was analyzed together with its pattern (locoregional, distant or mixed) and the frequency of disease-free patients at the end of the follow-ups. Four types of survival were studied (Table S1, Supplementary Materials). Overall survival, measured from the date of surgery to death from any cause, or censored at the last follow-up. Cancer-specific survival, considering only lung cancer deaths as an event. Recurrence-free survival, considering the occurrence of recurrence as an event. Disease-free survival, considering death from any cause or presence of disease at the last follow-up as an event. In the last case, patients who had a recurrence, but are treated and do not have disease at the last follow-up, are considered censored.

### 2.5. Statistical Analysis

A 2:1 propensity score (lobectomy:segmentectomy) paired with the nearest-neighbor method was performed to correct for the selection bias by indication that occurs in the choice of lung resection type. The aim was for the two groups to be balanced with respect to variables that were confounding factors and that could alter the relationship between the type of resection and the outcome analyzed. These factors were detected by observing those variables with significant differences between the groups or with significant influence on survival. These variables were subsequently used to obtain the propensity score to match patients and thus try to correct for selection bias. For the study of significant differences between groups, the *t*-test for continuous values and Fisher’s exact for categorical values were used. For the influence on survival, univariate Cox regression was used. For each type of survival, a different Cox analysis was performed, and the propensity score was obtained by choosing the variables derived from this analysis. In the case of overall survival, the variables selected were age, gender, smoking, FEV1, DLCO, tumor characteristics (size, SUVmax, location and radiological density), histological type, pN, number of lymph nodes in the lymphadenectomy and comorbidities such as alcoholism, HBP, DM, ischemic heart disease and peripheral vascular disease. In 4 of these variables (FEV1, DLCO, SUVmax of the tumor and number of lymph nodes) there were missing values, so two analyses were performed. One with all patients (eliminating variables with missing values), and another with all variables (eliminating patients with incomplete variables). The results of the former are depicted in the main manuscript and the latter in the Supplementary Materials.

After propensity score matching, the frequency of recurrence with its pattern and disease-free frequency were compared between the two groups with Fisher’s exact test. The different survival rates were compared by Kaplan-Meier and log-rank tests. Following the implementation of the propensity score, comparable groups were obtained, thereby enabling the execution of comparisons without the incorporation of additional covariates. The behavior of these covariates was constrained to be equivalent within the two groups.

R software (version 4.4.1, 14 June 2024), MatchIt library, was used.

## 3. Results

Figure 1 shows the patients included and excluded in this study. The types of segmentectomies are presented in Table S2 (Supplementary Materials). After propensity score matching, there remained 166 lobectomies and 83 segmentectomies in the overall survival analysis and the sample with all patients (Figure 1).

The characteristics of the patients are shown in Table 1. Significant differences between groups disappeared after matching.

Recurrence data were collected from 160 patients in the lobectomy group and 80 in the segmentectomy group. They presented recurrence in 38 and 19 patients respectively, a frequency of 23.7% in both groups. Table 2 shows the types of recurrence in each group. Among the patients with recurrence, the most frequent type in the lobectomy group was distant recurrence, with the addition of mixed one20 (52.6%), and the locoregional in the segmentectomy group 13 (68.4%). In the lobectomy group, 18 (11.2%) patients presented isolated locoregional recurrence, compared to 13 (16.2%) in the segmentectomy group (*p* = 0.309). Distant recurrence, also taking into account the mixed recurrence, occurred in 20 (12.5%) in the lobectomy group and 6 (7.5%) in the segmentectomy group (*p* = 0.278). At the end of the follow-ups, 108 (67.5%) patients in the lobectomy group were alive without disease, and 62 (77.5%) in the segmentectomy group (*p* = 0.132).

**Figure 2:**
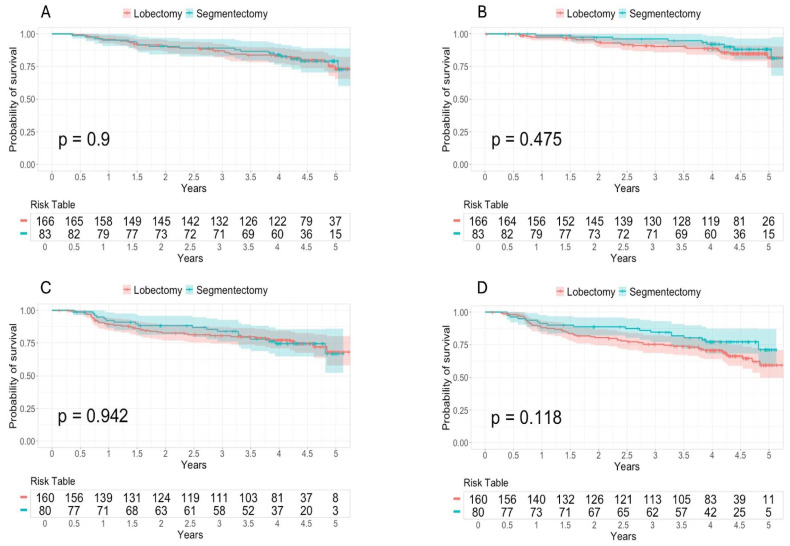
(**A**) Kaplan-Meier curves of the comparison of overall survival between lobectomy and segmentectomy after propensity score matching with the sample with all patients. The 95% confidence intervals and *p*-value corresponding to the log-rank test are shown. (**B**) Kaplan-Meier curves of the comparison of cancer-specific survival between lobectomy and segmentectomy after propensity score matching with the all-patient sample. The 95% confidence intervals and *p*-value corresponding to the log-rank test are shown. (**C**) Kaplan-Meier curves of the comparison of recurrence-free survival between lobectomy and segmentectomy after propensity score matching with the sample with all patients. The 95% confidence intervals and *p*-value corresponding to the log-rank test are shown. (**D**) Kaplan-Meier curves of the comparison of disease-free survival between lobectomy and segmentectomy after propensity score matching with the sample with all patients. The 95% confidence intervals and *p*-value corresponding to the log-rank test are shown.

There was no difference in overall survival (*p* = 0.950, Figure 2A), cancer-specific survival (*p* = 0.475, Figure 2B), recurrence-free survival (*p* = 0.942, Figure 2C) or disease-free survival (*p* = 0.118, Figure 2D). Overall survival at 2, 3 and 5 years was 90.3% (95% CI: 85.8–94.9%), 86.4% (95% CI: 81.3–91.9%) and 73.5% (95% CI: 65.5–82.4%) in the lobectomy group versus 89.1% (95% CI: 82.7–96.1%), 87.9% (95% CI: 81.1–95.2%) and 73.1% (95% CI: 60.1–88.9%) in the segmentectomy group. The data for the different types of survival at 2, 3 and 5 years are shown in Table S3 (Supplementary Materials).

The same analysis was performed, taking all the variables used in the propensity score and eliminating patients with missing values, leaving 88 patients in the lobectomy group and 44 in the segmentectomy group for the overall survival analysis (cohort of samples with all variables). In summary, 88 lobectomies and 44 segmentectomies were selected following propensity score matching. The characteristics of the covariates are displayed in Table S4. The results of the survival analyses are shown in Figures S1–S4, where the results are consistent with those obtained with the cohort with all patients.

## 4. Discussion

In this real-life observational study, we analyzed the long-term outcomes in routine clinical practice of anatomic segmentectomy compared with lobectomy for the surgical treatment of c-stage IA non-small cell lung cancer. To isolate the effect of resection type and avoid selection bias, we matched patients by propensity score using variables that influenced outcomes or differed significantly between groups. We observed a similar recurrence rate, and there were no differences in any type of survival at 5 years.

Lobectomy has been the main indication for the treatment of lung cancer since the trial by Ginsberg et al. [1]. Subsequently, multiple observational studies found equivalent outcomes of anatomic segmentectomy in patients who are older, frail or have decreased lung function [2,3,11,12]. Trisegmentectomy of the left upper lobe has been shown to be equivalent in terms of recurrence and survival compared to lobectomy, even for tumors up to 3 cm [13,14]. In tumors with a predominant ground-glass appearance, the indicated type of resection is also a segmentectomy, regardless of size [15,16]. In patients with nodules detected in screening programs, sublobar resection has demonstrated similar survival results compared to lobectomy [17].

Aside from these special situations, the interest in sublobar resection as the primary indication has progressively increased [18]. Multiple observational studies found similar results in early stages [5,6,7,19,20,21,22]. However, in some cases lobectomy showed superiority [23,24]. Variations in sample characteristics may change the outcome direction [25]. Doubts about the indicated type of resection have decreased with the two clinical trials conducted in Japan and the United States, and they have promoted segmentectomy as the first option for peripheral tumors smaller than 2 cm and N0 [8,9,26].

The main problem is the risk of recurrence [1]. We found no difference in the overall recurrence frequency, but a non-significant difference in pattern, being more frequent the locoregional type in segmentectomies and in the distant type in lobectomies. As in other studies [1,8,20], isolated locoregional recurrence was higher in segmentectomies (16.2% vs. 11.2%), but did not reach statistical significance. Data on recurrence vary greatly depending on the cases included. In subcentimeter tumors, low percentages can be observed, even with wedge resections: around 10% overall and 3% locoregional [19]. The Japanese trial showed a significantly higher locoregional recurrence in segmentectomies (6.9% vs. 3.1%) [8]. In the US trial, however, no differences were found (13.4% in sublobar vs. 10% in lobectomy) [9]. Overall recurrence was different in both trials: around 30% in the US and 12% in Japan. In the US trial, 59% of the sublobar resections were wedge resections and about 60% of the tumors were adenocarcinoma; whereas, in the Japanese trial, only anatomic segmentectomies were performed, and 90% of the tumors were adenocarcinoma. In our case, recurrence data more closely resemble the US trial data. In contrast to this trial, in our study only anatomic segmentectomies were included in the sublobar group. Analyzing the characteristics of the patients included in the two clinical trials and our series, it appears that differences in the histologic type of tumors may be the main reason for the deviation in the recurrence rate. In the Japanese trial, 90% of the included cases were adenocarcinoma, of which many could have been of the lepidic subtype which has been shown to have a better prognosis [15,16]. On the contrary, both in the US trial and in our series, 40% were of a histologic type other than adenocarcinoma, some of them with a worse prognosis.

In both trials, intraoperative confirmation N0 was required to perform sublobar resection, and in the Japanese trial, the surgical margin was checked. These maneuvers are not always performed in routine clinical practice and must be kept in mind when assessing the results. Both the surgical margin and lymph node involvement are important risk factors for recurrence [27,28]. Okada et al. demonstrated that, in sublobar resection, locoregional recurrence can be low (4.9%) and inferior to results from a lobectomy [5]. Nevertheless, in observational studies where there is no record of such verifications being performed, the recurrence rate tends to be higher [20,29]. In the study by Subramanian et al., similar in structure to ours, with propensity score adjustment, they observed a significantly higher risk of recurrence in sublobar resection [20]. Notably, out of 333 patients in the sublobar group, only 48 underwent anatomic segmentectomy. They did not provide data on the pattern of recurrence or radiological density, which are important for a correct assessment [29].

The main objective of surgical treatment is to increase survival. In our study, overall, cancer-specific, recurrence-free and disease-free survival were similar in both types of resections, with overall survival around 75% at 5 years. In the Japanese trial, segmentectomy had superior overall survival, over 90% at 5 years [8]. In the US trial, neither overall nor disease-free survival was inferior in the sublobar group [9]. The overall survival percentages were lower than the Japanese study and similar to ours. The differences in tumor type explained above may account for these differences. Both clinical trials conclude that sublobar resection may be the surgical treatment of first choice in peripheral tumors, N0 and smaller than 2 cm. They speculate that performing a more conservative surgery may allow a more aggressive treatment of future medical pathologies or second neoplasms favoring a longer survival [8].

Clinical trials provide higher-quality scientific evidence, but it is a highly selected population with extensive controls, such as intraoperative margin and node review, which may differ from routine clinical practice. Observational studies better reflect the outcomes obtained in real life [22]. In those performed with national databases, there are conflicting results due to the different samples studied [7,20,23,24]. Using the US Surveillance, Epidemiology and End Results (SEER) database, Dai et al. observed better overall and cancer-specific survival from lobectomy [23], while Zhao et al. observed no difference [7]. The same phenomenon occurs with the US National Cancer Database (NCDB), where Khullar et al. observed better overall survival from lobectomies [24], but Subramanian et al. found no such differences [20]. The most recent evidence comes from two studies with the SEER database and the French national database [21,22]. The first study observed no differences between lobectomies and segmentectomies in overall survival at 5 years [21], but in the second study survival was lower in the segmentectomy group, which leads to the conclusion that a lobectomy should be the first option [22]. The great strength of both studies is the large number of patients included, but they present methodological deficiencies that should be taken into account. Both lack data on recurrence, and the SEER database does not have fundamental confounding factors to assess survival such as comorbidity or respiratory function tests [21], In the French study, the mean follow-up was 38 months [22], insufficient to assess 5-year survival, especially considering that segmentectomies confer longer-term protection [8].

### Limitations

First, those inherent to using a database not designed for this study so that we do not have some variables for calculating the propensity score. This is a problem common to all studies performed with national databases. Being a multicenter study, the diagnostic and treatment management is not homogeneous. It is not reflected in the database whether there was intraoperative assessment of nodes and surgical margins as required in major clinical trials [8,9]. However, this is not the usual practice, and our data reflect the results obtained in real life, when the strict controls of clinical trials are not met. The number of segmentectomies included was not high, which reduces the statistical power and may lead to Type II errors. The mean follow-up period was 51 months, so some late recurrences beyond this timeframe may not have been captured. Nevertheless, we think it is enough to assess long-term results, longer than other studies from national registries [22].

Strengths can also be found in our work. It is a prospective database with a high level of recruitment and auditing of the information entered [10]. The patients were recruited in a short period of time (15 months), so that variations in time-dependent risk factors, such as perioperative treatment schedules, have less influence on the outcomes. It has key prognostic factors for adjustment such as comorbidity, functional tests, SUVmax, radiological density or number of nodes. Finally, the data are highly representative of standard clinical practice.

## 5. Conclusions

In the surgical treatment of clinical stage IA lung cancer, the frequency of tumor recurrence is similar in lobectomies and segmentectomies, but the pattern of recurrence is different, with locoregional recurrence being more frequent in segmentectomies, and distant recurrence in lobectomies. The long-term survival results of anatomic segmentectomies in routine clinical practice compared to lobectomies are equivalent. In view of these results, anatomic segmentectomy appears to be a valid alternative for clinical stage IA lung cancer in select patients. However, the trend toward increased locoregional recurrence suggests careful patient selection is necessary, and further randomized trials with standardized surgical protocols are needed to confirm these findings.

## Figures and Tables

**Table 1:** Characteristics of patients divided into the lobectomy and segmentectomy groups before and after propensity score matching.

	Pre-Matching Groups			Post-Matching Groups		
Characteristics	Lobectomy (*n* = 1004)	Segmentectomy (*n* = 83)	*p*-Value	Lobectomy (*n* = 166)	Segmentectomy (*n* = 83)	*p*-Value
Age (years)	**65.1 (10.1)**	**68.1 (8.2)**	**0.002**	**67.7 (9.5)**	**68.1 (8.2)**	**0.705**
Gender (M)	669 (66.6)	52 (62.6)	0.470	103 (62.1)	52 (62.6)	1
Smoking			0.149			0.941
Smoker	317 (31.6)	23 (27.7)		52 (31.3)	23 (27.7)	
Ex-smoker > 12 m	130 (12.9)	5 (6.0)		7 (4.2)	5 (6.0)	
Ex-smoker 1–12 m	394 (39.2)	42 (50.6)		79 (47.6)	42 (50.6)	
Never smoker	147 (14.6)	11 (13.2)		24 (14.5)	11 (13.2)	
Unknown	16 (1.6)	2 (2.4)		4 (2.4)	2 (2.4)	
HBP	458 (45.6)	46 (55.4)	0.087	92 (55.4)	46 (55.4)	1
DM	182 (18.1)	16 (19.3)	0.768	35 (21.1)	16 (19.3)	0.868
Cardiac failure	23 (2.3)	3 (3.6)	0.442	7 (4.2)	3 (3.6)	1
Ischemic heart disease	85 (8.5)	11 (13.2)	0.156	22 (13.2)	11 (13.2)	1
Arrhythmia	70 (6.9)	9 (10.8)	0.187	13 (7.8)	9 (10.8)	0.479
Peripheral vascular disease	104 (10.4)	9 (10.8)	0.852	22 (13.2)	9 (10.8)	0.686
Creatinine > 2	32 (3.2)	2 (2.4)	1	11 (6.6)	2 (2.4)	0.229
Previous cardiac surgery	16 (1.6)	1 (1.2)	1	4 (2.4)	1 (1.2)	0.667
Alcoholism	**79 (7.9)**	**1 (1.2)**	**0.025**	**1 (0.6)**	**1 (1.2)**	**1**
Liver failure	17 (1.7)	0 (0)	0.634	2 (1.2)	0 (0)	0.554
**Tumor size (mm)**	**17.8 (7.9)**	**16.0 (6.1)**	**0.014**	**16.4 (8.1)**	**16.0 (6.1)**	**0.686**
Radiological tumor density			0.448			0.433
Mixed	170 (16.9)	17 (20.5)		42 (25.3)	17 (20.5)	
Solid	834 (83.1)	66 (79.5)		124 (74.7)	66 (79.5)	
**Tumor location**			**0.002**			**0.839**
Peripheral	**718 (71.5)**	**72 (86.8)**		**146 (87.9)**	**72 (86.8)**	
Central	**286 (28.5)**	**11 (13.2)**		**20 (12.1)**	**11 (13.2)**	
Histological type			0.549			0.921
Adenocarcinoma	608 (60.6)	46 (55.4)		91 (54.8)	46 (55.4)	
Squamous	240 (23.9)	23 (27.7)		47 (28.3)	23 (27.7)	
Other	156 (15.5)	14 (16.9)		28 (16.9)	14 (16.9)	
Surgical approach			0.530			0.653
Open	290 (28.9)	21 (25.3)		48 (28.9)	21 (25.3)	
VATS	714 (71.1)	62 (74.7)		118 (71.1)	62 (74.7)	
pN			0.483			0.937
pN0	880 (87.6)	76 (91.6)		148 (89.2)	76 (91.6)	
pN1	61 (6.1)	2 (2.4)		5 (3.0)	2 (2.4)	
pN2	62 (6.2)	5 (6.0)		13 (7.8)	5 (6.0)	
pNx	1 (0.1)	0 (0)		(0)	0 (0)	

Data are shown with the mean and standard deviations in parentheses for continuous variables or absolute numbers and percentage in parentheses for categorical variables. Variables with significant differences between groups before matching are marked in bold. DM: diabetes mellitus; HBP: high blood pressure; M: male; pN: pathological lymph node staging; VATS: video-assisted thoracic surgery.

**Table 2:** Types of recurrence in the lobectomy and segmentectomy groups after propensity score matching.

Type of Recurrence	Lobectomy (*n* = 160)	Segmentectomy (*n* = 80)	*p*-Value
Locoregional	18 (11.2)	13 (16.2)	0.309
Distant	13 (8.2)	4 (5.0)	0.436
Mixed	7 (4.4)	2 (2.5)	0.722

Data are shown with absolute number and percentage in parentheses.

## Data Availability

The data underlying this article will be shared on reasonable request to the corresponding author.

## Supplementary Materials

The following supporting information can be downloaded at: https://www.mdpi.com/article/10.3390/jcm14072267/s1. Table S1: Types of survival analyzed in the study; Table S2: Types of segmentectomy (*n* = 83); Table S3: Survival of patients divided into the lobectomy and segmentectomy groups after propensity score matching in the sample with all patients; Table S4: Survival of patients divided into the lobectomy and segmentectomy groups after matching by propensity score in the sample with all variables; Figure S1: Kaplan-Meier curves of the comparison of overall survival between lobectomy and segmentectomy after propensity score matching with the sample with all variables. The 95% confidence intervals and *p*-value corresponding to the Log-rank test are shown; Figure S2: Kaplan-Meier curves of the comparison of cancer-specific survival between lobectomy and segmentectomy after propensity score matching with the sample with all variables. The 95% confidence intervals and *p*-value corresponding to the log-rank test are shown; Figure S3: Kaplan-Meier curves of the comparison of recurrence-free survival between lobectomy and segmentectomy after propensity score matching with the sample with all variables. The 95% confidence intervals and *p*-value corresponding to the Log-rank test are shown; Figure S4: Kaplan-Meier curves of the comparison of disease-free survival between lobectomy and segmentectomy after propensity score matching with the sample with all variables. The 95% confidence intervals and *p*-value corresponding to the Log-rank test are shown.

## Abbreviations

The following abbreviations are used in this manuscript:

DLCO	diffusion capacity of carbon monoxide.
DM	diabetes mellitus.
FEV1	forced expiratory volume in 1 s.
GEVATS	Spanish Group of Video-Assisted Surgery.
HBP	high blood pressure.
SECT	Spanish Society of Thoracic Surgery.
SEER	Surveillance, Epidemiology and End Results.
SUVmax	maximum standardized uptake value.
CT	computed tomography.
VATS	video-assisted thoracic surgery.
