# Supplementary material for: Long-Term Results of Segmentectomy vs. Lobectomy for c-Stage IA Lung Cancer: A Real-Life Study with a Propensity Score Analysis Based on a National Cohort
Source: J Clin Med. 2025 Mar 26;14(7):2267. doi: 10.3390/jcm14072267 (PMC11989587; doi:10.3390/jcm14072267)
Supplement: Supplementary file 1 [file jcm-14-02267-s001.zip › jcm-3504486-supplementary.pdf]

Table S1. Types of survival analyzed in the study.

	Event	Time	Censored	Censoring time
Overall survival	Death from any cause	Time to death	Alive at the last follow-up	Last day of follow-up
Cancer-specific survival	Death from lung cancer	Time to death from lung cancer	Alive at last follow-up or died of another cause before completion of the study	Last day of follow-up or time to death from other causes
Recurrence-free survival	Tumor recurrence	Time to recurrence	Absence of recurrence or death from a cause other than lung cancer	Last day of follow-up or time to death from other causes
Disease-free survival	Death from any cause or presence of disease at last follow-up	Time to recurrence or death	Alive at the last follow-up and without disease	Last day of follow-up

Table S2. Types of segmentectomy (n = 83).

Segmentectomy type	n (%)
Trisegmentectomy (S1+2-3 LUL)	31 (37.3)
S6 LLL	14 (16.9)
Lingulectomy (S4-5 LUL)	10 (12.0)
S6 RLL	5 (6.0)
S1 RUL	4 (4.8)
S2 RUL	3 (3.6)
S1+S2 RUL	2 (2.4)
S1 LUL	2 (2.4)
S1+2 LUL	2 (2.4)
S3 RUL	2 (2.4)
Basal pyramid LLL	2 (2.4)
Basal pyramid RLL	1 (1.2)
S2 LUL	1 (1.2)
S3 LUL	1 (1.2)
S7 RLL	1 (1.2)
S8-9-10 RLL	1 (1.2)
Unknown	1 (1.2)

LLL: left lower lobectomy; LUL: left upper lobectomy; RLL: right lower lobectomy; RUL: right upper lobectomy; S: segmentectomy.

Table S3. Survival of patients divided into the lobectomy and segmentectomy groups after propensity score matching in the sample with all patients.

Survival type	Lobectomy<br>% (CI 95%)	Segmentectomy<br>% (CI 95%)	P-value
Overall survival<br>(166 vs 83 patients)			0.950
2 years	90.3 (85.8 – 94.9)	89.1 (82.7 – 96.1)	
3 years	86.4 (81.3 – 91.9)	87.9 (81.1 – 95.2)	
5 years	73.5 (65.5 – 82.4)	73.1 (60.1 – 88.9)	
Cancer-specific survival<br>(166 vs 83 patients)			0.475
2 years	93.0 (89.1 – 97.1)	96.1 (91.9 – 100)	
3 years	89.6 (85.0 – 94.6)	94.8 (89.9 – 99.9)	
5 years	81.9 (74.1 – 90.4)	81.4 (67.9 – 97.5)	
Recurrence-free survival<br>(160 vs 80 patients)			0.942
2 years	82.0 (76.1 – 88.3)	86.9 (79.6 – 94.8)	
3 years	79.9 (73.8 – 86.5)	82.5 (74.2 – 91.6)	
5 years	68.3 (58.1 – 80.3)	67.0 (52.3 – 86.0)	
Disease-free survival<br>(160 vs 80 patients)			0.118
2 years	79.8 (73.8 – 86.3)	97.4 (80.4 – 95.0)	
3 years	74.6 (68.1 – 81.7)	83.2 (75.3 – 92.0)	
5 years	59.5 (49.9 – 70.9)	71.3 (58.4 – 87.1)	

Table S4. Survival of patients divided into the lobectomy and segmentectomy groups after matching by propensity score in the sample with all variables.

Survival type	Lobectomy<br>% (CI 95%)	Segmentectomy<br>% (CI 95%)	P-value
Overall survival<br>(88 vs 44 patients)			0.483
2 years	88.4 (81.9 – 95.4)	90.8 (82.6 – 99.8)	
3 years	83.6 (76.1 – 91.9)	88.3 (79.2 – 98.5)	
5 years	69.3 (57.4 – 83.8)	*	
Cancer-specific survival<br>(88 vs 44 patients)			0.890
2 years	92.6 (87.1 – 98.5)	95.1 (88.7 – 100)	
3 years	86.9 (79.6 – 94.9)	92.5 (84.8 – 100)	
5 years	*	*	
Recurrence-free survival<br>(86 vs 43 patients)			0.742
2 years	86.0 (78.7 – 94.1)	87.7 (78.2 – 98.4)	
3 years	83.0 (75.0 – 91.9)	84.8 (74.3 – 96.9)	
5 years	75.6 (63.3 – 90.1)	*	
Disease-free survival<br>(86 vs 43 patients)			0.351
2 years	81.4 (73.4 – 90.4)	88.0 (78.7 – 98.5)	
3 years	77.4 (68.7 – 87.2)	85.2 (74.9 – 96.9)	
5 years	65.6 (53.1 – 80.9)	*	

*There was insufficient follow-up time in the sample to calculate the probability of survival.

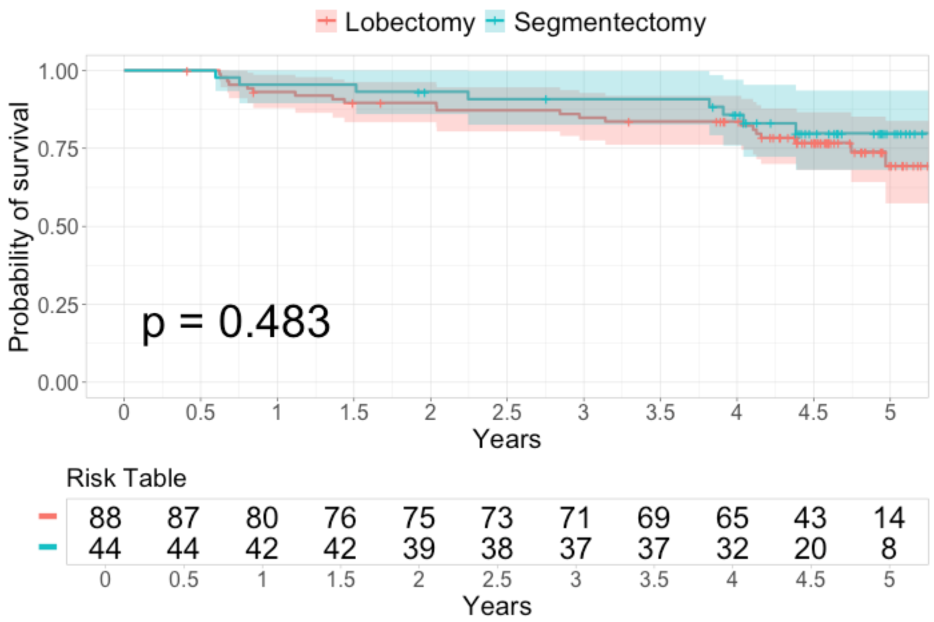

Fiure S1. Kaplan-Meier curves of the comparison of overall survival between lobectomy and segmentectomy after propensity score matching with the sample with all variables. The 95% confidence intervals and P-value corresponding to the Log-rank test are shown.

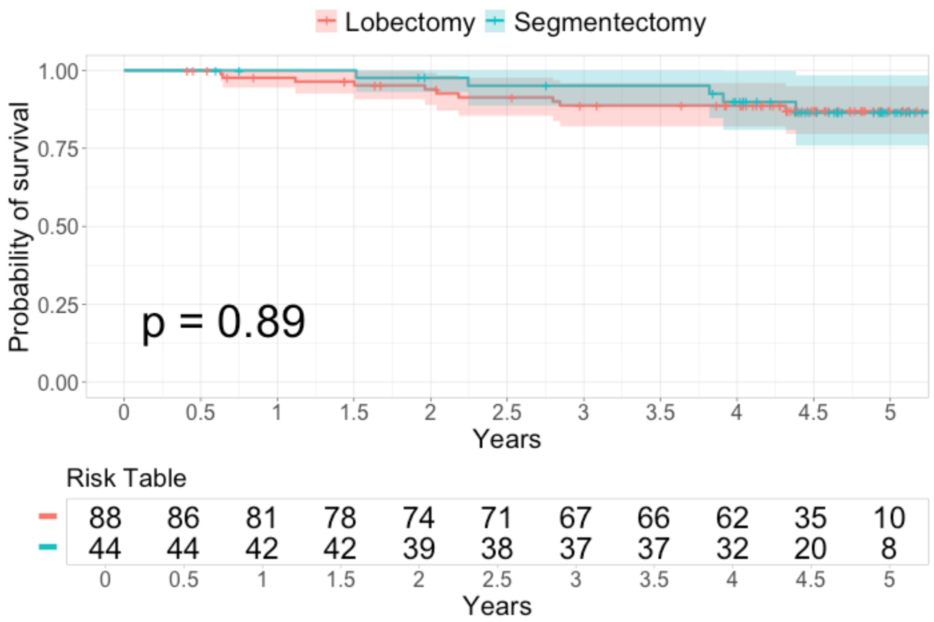

Figure S2. Kaplan-Meier curves of the comparison of cancer-specific survival between lobectomy and segmentectomy after propensity score matching with the sample with all variables. The 95% confidence intervals and P-value corresponding to the log-rank test are shown.

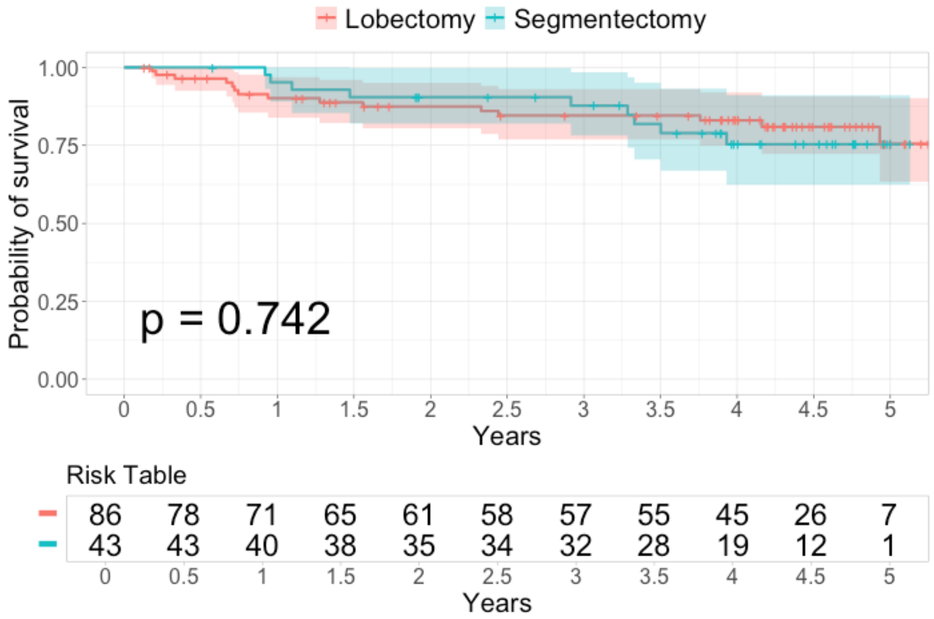

Figuer S3. Kaplan-Meier curves of the comparison of recurrence-free survival between lobectomy and segmentectomy after propensity score matching with the sample with all variables. The 95% confidence intervals and P-value corresponding to the Log-rank test are shown.

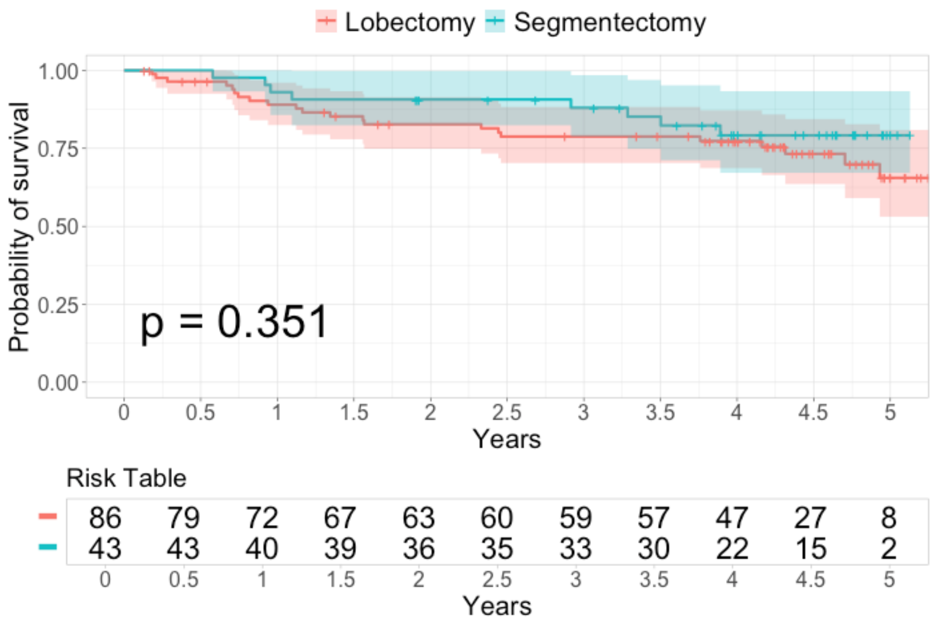

Figure S4. Kaplan-Meier curves of the comparison of disease-free survival between lobectomy and segmentectomy after propensity score matching with the sample with all variables. The 95% confidence intervals and P-value corresponding to the Log-rank test are shown.
